# Empathy, caring and compassion: Toward a Freudian critique of nursing work

**DOI:** 10.1111/nup.12399

**Published:** 2022-06-19

**Authors:** Michael Traynor

**Affiliations:** ^1^ Centre for Critical Research in Nursing and Midwifery Faculty of Health, Social Care and Education, Middlesex University London UK

**Keywords:** care, hermeneutics, nursing, psychoanalysis, Sigmund Freud

## Abstract

The aim of this paper is to summarize key psychoanalytic concepts first developed by Sigmund Freud and apply them to a critical exploration of three terms that are central to nursing's self‐image—empathy, caring, and compassion. Looking to Menzies‐Lyth's work, I suggest that the nurse's strong identification as a carer can be understood as a fantasy of being the one who is cared for; critiques by Freud and others of empathy point to the possibility of it being, in reality, a form of projective identification; reading Lacan and Žižek, I propose that repeated research into caring and repeated complaint about barriers to caring can be understood as manifestations of the death drive first posited by Freud. I conclude that psychoanalytic insights suggest that caring roles can raise profoundly ambivalent issues for those who care but they can also point the way to freedom from painful and self‐destructive symptoms inherent in such work.

## 1 INTRODUCTION

The aim of this paper is to critically explore terms that are central to nursing's self‐image—empathy, caring, and compassion. This paper is part of a larger project investigating what the thinking of the three “masters of suspicion,” Marx, Freud, and Nietzsche, might contribute to a new understanding—a critique—of nursing.

I start by examining the chain of signifiers (Derrida, 1982; Lacan, 1977) that, along with “autonomy,” examined in a previous paper (Traynor, 2019), we might consider as close to the core of nursing's self‐image: these are empathy, caring, and compassion. I then summarize some of the concepts and theories that Freud developed, setting out a background for three of these that I will draw on: the unconscious, infantile sexuality, and the death drive. I will also summarize key critiques of Freud and point to the development of Freudian concepts by subsequent theorists. I will then return to psychoanalytic inquiries into nursing work, as well as Freud's response to the biblical injunction to “love your neighbour,” to see these key signifiers for nursing in a new light. My aim is to acquaint the unfamiliar reader with some of Freud's ideas and give an account of how they might be applied to thinking about nursing.

## 2 FREUD AND THE THREE MASTERS OF SUSPICION

Philosopher Paul Ricoeur is credited with describing three European thinkers as “masters of suspicion.” He considered Karl Marx (1818–1883), a political economist and sociologist, the philosopher Friedrich Nietzsche (1844–1900), and Sigmund Freud (1856–1939), the founder of psychoanalysis shaped the intellectual mood of the 20th century. Working in different areas, each of them, according to Ricoeur, “sought to unmask, demystify, and expose the real from the apparent… not only by means of a ‘destructive’ critique but by the invention of an art of interpreting” (Ricoeur, 1988 p. 194). Each exposed what we might call false consciousness. Their ideas have pulled the rug out from under many of Western society's comforting cultural and personal certainties. Out of their work has developed a broad and fertile range of critical theoretical studies that include aspects of structuralism and poststructuralism. Workers in literary studies, cultural theory, anthropology, and legal studies, as well as those within political science, philosophy, and psychoanalysis, are likely to draw from the theories and impulses of the three “masters” and those who have developed their work. Notable among them are cultural theorist Slavoj Žižek whose writing on contemporary political events, brings together theory from Marx, continental philosophy, and Lacanian psychoanalysis. In work on literature Jonathan Culler (Culler, 1983), Julia Kristeva (McAfee, 2004), Eric Santner (Zizek et al., 2005), and others explore and critique writing with approaches imbued with this thinking, sometimes explicitly in the form of psychoanalytic readings of the text. In a kind of loose lineage, we might identify more familiar figures to nurses such as Michel Foucault, Edward Said, or the theorists of the Frankfurt School as developing a critical field that the contemporary workers just identified continue to advance. And while some or all of these writers might owe intellectual debts to the “masters,” that debt may well take the form of robust critiques of their work. Some individual concepts in this theoretical field are explicit developments of earlier terms. For example, Marxist Louis Althusser, as well as the members of the Frankfurt School, looked to Freudian theories of the unconscious to develop novel explanations for the mechanisms of ideology (Althusser, 2014), a concept set out by Marx and Engels as a key component to the operation of capitalism (Marx & Engels, 1998 [1845]). In summary, psychoanalytic work has gone beyond the clinic.

## 3 A SHORT NOTE ON METHOD

While writing this essay a short text came to my notice that both guides and challenges the work. It is taken from an editorial preface written by Slavoj Žižek to a series of critical books entitled “short circuits” (see https://mitpress.mit.edu/books/series/short-circuits):

A short circuit occurs when there is a faulty connection in the network – faulty, of course, from the standpoint of the network's smooth functioning. Is not the shock of short‐circuiting, therefore, one of the best metaphors for a critical reading? Is not one of the most effective critical procedures to cross wires that do not usually touch: to take a major classic (text, author, notion) and read it in a short‐circuiting way, through the lens of a “minor” author, text, or conceptual apparatus (“minor” should be understood here in Deleuze's sense: not “of lesser quality,” but marginalized, disavowed by the hegemonic ideology…)? If the minor reference is well chosen, such a procedure can lead to insights that completely shatter and undermine our common perceptions. This is… what Freud and Nietzsche did with morality… the aim of such an approach is… the inherent decentring of the interpreted text, which brings to light its “unthought,” its disavowed presuppositions and consequences.

In this essay, perhaps counterintuitively, the major classic takes the form of influential nursing texts about empathy, caring, and compassion. The “marginalized” texts are those of Freud and workers in his tradition. We could argue that this psychoanalytic writing is indeed marginalized from mainstream scientific culture (see later Section 4.3) and we could also see it as pushed to the margins of “mainstream” nursing theory. Nursing's response to Menzies‐Lyth's writing on the organization of nursing work, discussed later, was largely one of marginalization.

### 3.1 Empathy, caring, and compassion as signifiers in nursing

Before turning to psychoanalytic argument, I would like to briefly explore the contexts in which these terms, empathy, caring, and compassion, have been developed in nursing and which groups have used them, and to the achievement of which ends. The key point of relevance to my main argument is that in professional discourse, these qualities are often described as blocked in their expression by nurses in the workplace. The approach taken here owes something to Foucault's idea of archaeology of a field of knowledge (Foucault, 1989). I will return to psychoanalytic concepts to explore the way that individual nurses might unconsciously engage with this aspect of professional discourse.

I suggest that the use of these terms cannot be separated from claims for professional nursing identity and value. Their contemporary use often refers back to an earlier time, real or (more likely) imagined, in which their expression was natural and unimpeded in nursing. Nelson argues that while the contemporary drive to express and promote humanistic ideals in nursing can be understood as a secularization of an older Christian imperative within nursing work (Nelson, 1995), the imprint of duty and servitude remains. Some claim Florence Nightingale as a champion of empathy, though not necessarily expressed in religious terms (Maben et al., 2009) and many have presented empathetic, compassionate caring as nursing's distinguishing, if not unique, feature within health service delivery. Benner and Wrubel's The Primacy of Caring (1989) and other foundational texts published in the 1980s present nursing practice as embodying humanistic principles (Paterson & Zderad, 1976). Much of this literature describes how the nurse consciously enters into an empathetic and therapeutic relationship with patients. This can allow the nurse to deliver more individualized care, however, this literature claims that to do this is to also participate in the grander project of “the development of human potential“ (Paterson & Zderad, 1976, p. 93) an elevation both of caring and of the profession. In this and other writing from the period, as well as more recent writing, a humanistic basis to nursing practice is almost always discussed in contrast to forces outside nursing constraining its proper expression (Parse, 1999; Playle, 1995; Wong Woon, 2004). Some contrast “the art of caring” with a managerialist focus on counting the countable “second‐order” activities whose apparent concreteness renders caring, by contrast, invisible (Maben et al., 2009). So the presentation of empathy, caring, and compassion very often comes coupled with the threat of their opposite that is always in danger of overwhelming their expression by nurses. In ideological terms, this has the effect of keeping the promise of its value always alive. In psychoanalytic terms, the repetition of failure (to be properly empathetic) alerts us to the operation of the death drive, a proposition that I will return to later.

Some writers have taken a more skeptical approach to nursing's long‐standing preoccupation with caring yet have discovered the same blocked progress. Paley's (2001) approach to nursing texts which purport to investigate and understand the nature of “caring” identifies a potentially endless repetition of thesaurus‐like terms that many authors present as analysis. Researchers of caring have produced extensive heterogeneous lists of what their participants—nurses—have said about caring. These lists bear a similarity, Paley notes, to the exhaustive encyclopedias that Foucault identifies as characterizing premodern knowledge (Foucault, 1972). Paley (2001, p. 195) uses the terms “endlessness and uselessness” regarding this project. There is a circularity in the use of these signifiers of caring that appears to lack an exit point.

To summarize, empathy, caring and compassion can be seen as members of a chain of signifiers signifying personal characteristics that are the “possession” of the nurse. Understanding them as “second‐order signifiers” (Barthes, 1972) allows us to interpret them as signifying a value that is fundamental to nursing's professional identity and ideology. Repeated research into the nature of caring is, I would like to suggest, an endless failure that performs the function of keeping caring alive as a transcendent value by the repetition of the mock attempt to understand it. In the next section, I set out a more detailed examination of these signifiers from the perspective of Freud's skepticism.

## 4 SIGMUND FREUD

Sigmund Freud was an Austrian neurologist and widely considered to be the “father” of psychoanalysis. Because of Freud's position—as a doctor attempting to treat patients with sometimes incapacitating problems—his focus was on psychopathology, literally the study of the suffering of the soul. Often this suffering, he discovered, was related to traumatic events or unresolved tensions in childhood. From his practice, Freud developed his well‐known “talking cure” and an elaborate range of theories that he revised and refined throughout his life. However, Freud's field of analysis was far wider than the sufferings of the small stream of individual patients who came to lie on his couch in Vienna and London during the late 19th and early decades of the 20th century. Later in his career, Freud turned his interpretive approach to whole civilizations alongside the dreams of his patients and produced accounts—of both—that were counterintuitive and often unsettling. His suspicion was and his work seems to show that, in simple terms, there is more meaning to any statement than first meets the eye and than is intended by its author. It is his practice of “reading” or interpretation that accounts for Ricoeur's label. His interpretations may appear speculative and fail to meet particular standards of falsifiability (Popper, 2002 [1959]) but they emerge from and contribute to the elaboration of a body of meticulous theory. They invite, or rather challenge the listener—or patient—to look at things radically differently. It is the *possibility* of a theoretically driven, counterintuitive, and confronting reading of the taken for granted and cherished that I want to offer to the reader of this article.

### 4.1 Workers in a Freudian tradition

A common categorization of the development of Freud's thinking after his death describes three rival strands: the British school of object‐relations, the ego psychology of the United States, and French structuralist language‐based work (Hopkins, 1998). The work of Carl Jung, once a collaborator of Freud, is generally considered to form a different tradition and will not be considered in this paper. All three move away from Freud's biological explanations for the structure and activities of the human psyche. Object‐relations analysts focus their attention on the mother‐infant relationship as a model for subsequent adult relationships and emphasize the phantasy life of infants as the site of powerful forces and anxieties that can re‐emerge in adulthood. Isobel Menzies Lyth's well‐known analysis of organizational problems with the training of nurses undertaken while she worked at the Tavistock Institute in London is work within this tradition (Menzies, 1960). Ego psychology developed principally in the United States as a result of the migration of a number of European analysts during and after the Second World War. While for Freud even the ego, the most rational and conscious part of the psyche, comprised unconscious elements, ego psychologists focussed on the possibility of unity within the patient's ego. They attempted, in analysis, to strengthen the ego's innate ability to unify other aspects of the psyche and to adapt to its environment.

The key figure associated with the third strand is French psychoanalyst Jacques Lacan (1901–1981). He brought to bear elements of structuralism—Levi‐Strauss' anthropological analyses (Lévi‐Strauss, 2005 [1978]) and Saussure's linguistics (De Saussure, 1974)—onto Freud's theories and concepts with a strong focus on the powerful organizing work of language on the human subject. Because his theories are intertwined with philosophical and structuralist and post‐structuralist ideas, they remain a fertile ground for subsequent thinkers within the field of psychoanalysis and beyond. I will return to Lacan's development of some of Freud's theoretical work in the next section.

### 4.2 Three theories from Freud

Among a great many and often now well‐known Freudian propositions three, controversial at the time of their development and publication, can be considered central to his legacy: infantile sexuality, the unconscious, and the “death drive.” Because I plan to draw on these when I apply Freud's thinking to a critique of aspects of nursing, I will set them out now.

In European societies that prized and protected the innocence of childhood, Freud's suggestion that the pleasures experienced by the baby and young child, and provoked by its caregiver (for Freud this is the mother) are libidinous or erotic in character, came as scandalous. Freud claimed that the young infant is “polymorphously perverse,” able to gain sexual pleasure from any part of the body but that during development this pleasure becomes focused on specific body areas associated with different phases of development, the oral, anal, and genital phases (Freud, 1953a). For example, the sucking of breastfeeding originally serves the purpose of taking in sustenance but because of its pleasurable character, the pleasure later becomes separate from that function, at which time its sole purpose is pleasure, to be seen in the thumb‐sucking of the baby and child. Freud sees this development as the source of adult sexual pleasure. These infantile forms of sexuality became the basis for his proposed “Oedipus complex” first set out in 1899 (Freud, 1965), a term describing what he considered to be normal psychic development in terms of the child's renunciation of incestuous desires for its mother (his theory first concerned male infants) under the fantasized threat of castration by the father, toward whom the infant harbors murderous intent tempered with an awareness of the father's greater power. The successful “dissolution” of the Oedipus complex is achieved when the child is able to replace murderous rivalry with identification with the same‐sex parent. The element of this theory that I will take up later is the picture of infancy as a tumultuous time of psychic conflict involving terror and hate as well as love.

The second theory concerns the proposal and the elaboration of the notion of the unconscious. The unconscious, Freud argued, is a result of the continual repression of thoughts, ideas, and memories, often from childhood, that are too difficult or traumatic to process consciously. These disappear from the conscious mind but continue to be active and can reappear in consciousness in certain circumstances. In other words, we are unaware of them yet they influence our thoughts, emotions, and behavior in apparently irrational and possibly destructive ways. The work of the unconscious might be glimpsed through dreams, jokes, and slips of the tongue. Freud set out various ways of understanding the place and function of the unconscious, one of these being topological: the conscious, preconscious, and unconscious as regions of the human psyche. Later he proposed a different psychic topography of id, ego, and superego (Freud & Strachey, 1989). The id (literally in English the “it” – these Latinized translations being long‐established but controversial) is the entirely unconscious region of the psyche that operates on the so‐called pleasure principle and is the source of impulses and drives seeking immediate pleasure and gratification. The ego describes a broadly rational part of the psyche constrained by the principle of reality and struggling to control or modify the powerful and, in developmental terms, primitive drives of the id. The third part he labeled the superego, harshly critical, full of moral and societal imperatives, using guilt, anxiety, and a sense of inferiority as its modus operandi. Freud's model sees the ego as resorting to various forms of defenses when dealing with the demands of the id—denial, fantasy, regression, and sublimation among them. Freud described the ego as not being “master in its own house” (Nicht Herr im eigenen Haus) (Freud, 1953b). The human psyche as a divided site of continually unresolved yet hidden conflicting interests is a starkly different picture from the rational and unified self of the enlightenment philosophers (Adorno & Horkheimer, 1979).

The proposal of the “death drive” came late in Freud's work. He developed this aspect of his theory after years of observing that individuals appear to compulsively repeat painful or traumatic but familiar experiences. He noted this among his patients and others such as “shell‐shocked” First World War veterans or his grandson playing the game of “Fort/Da” – “gone/there” repeatedly throwing a toy (a bobbin on a string) out of sight which, he argued, restaged the distressing disappearance of his mother. Such observations led him to propose an alternative, opposing, and in some ways more fundamental drive to libido, his proposed basic life drive in humans. He looked to both biology and what might be called cosmic forces for an explanation. Biologically he proposed a mechanism of neutralization of drive excitation within the human mental apparatus and cosmically, he understood the death drive as ‘an urge in organic life to restore an earlier state of things' (Freud, 1984 [1920]), the inorganic state from which life originally emerged—hence the death in death drive. Subsequent thinkers working in Freud's tradition have generally rejected these foundations for understanding the psyche, some seeing Freud's insertion of teleology into the actually meaningless capacity of the psyche to disrupt itself as a significant mistake in his thinking (Lear, 2000). Jacques Lacan identified the “death drive” with an automatism that brings with it a certain enjoyment or satisfaction. He proposed that the drive satisfies itself by missing its object of desire (in diagrammatic terms looping around it and returning empty‐handed), rather than by obtaining it (Lacan, 1992). Slavoj Žižek, developing Lacan's idea further, proposed that the drive's true aim, which is masked, is to maintain the circular motion of repeatedly missing its object. The purpose of the drive, in this model, is to keep repeating:

…while the goal is the object around which the drive circulates, its true aim is the endless continuation of its circulation as such (Žižek, 2012, pp. 496–497)

(See Hook (2016) for a discussion of Žižek's writing on the death drive.) One helpful, in psychic terms, outcome of this continual failure is that the unattained lost object can be given and retain a heightened and even transcendental value. The life of empathy within bureaucratic health systems might be considered such an object for nurses.

### 4.3 Critiques of Freud and psychoanalysis

Both Freud individually and psychoanalysis as a practice have been roundly critiqued and criticized to such an extent that many today would see both as thoroughly discredited. Yet, as Thurschwell argues in her own introduction to critiques of Freud, “Like an endlessly recyclable horror‐film axe murderer, the more psychoanalysis is killed off, the more it comes back to haunt our culture” (Thurschwell, 2000, p. 113).

The critiques take three forms: critiques concerning a lack of scientific rigor; a feminist critique of Freud's view of gender and sexuality and an empiricist critique of his privileging of the apparently “inner” world of fantasy over history and concrete occurrence. There are in addition many ad hominem attacks on Freud—he was high on cocaine when he wrote much of his sloppiest work, for example—intended to strip his theories of any remnant of credibility (see Menand, 2017). Those who want to argue the case for the ax murderer have two options. One is to emphasize that current workers in the field of psychoanalytic studies and practice have developed Freud's work in a way that confronts and points beyond the cultural blind spots that afflicted the father of the discipline. The second is to claim that Freud has been misunderstood thoroughly and at times wilfully.

Critiques regarding the lack of a sound scientific basis for Freud's theories center around his development of general claims about the human psyche based largely on 12 inconsistent case studies of affluent Viennese that achieved uncertain outcomes (Grünbaum, 1985). Some claim that Freud should have compared his results with a number of “controls” (Colby, 1960), though it is hard to know what might have constituted psychiatric “treatment as usual” in 1890s Austria. Feminist critiques point to Freud's approach to understanding and making normative comments about, sexuality from a continually male perspective. The male is the norm from which unfathomable, complex, and duplicitous females depart (Irigaray, 1985). Feminists argue—it is not difficult—that Freud proposed and reinforced the claim for biological bases for what are largely social differences and inequalities between the sexes and their experiences (de Beauvoir, 1953). For a compelling account of some of Freud's more chauvinistic ideas about women and feminist critiques see (Gilman, 1971). The third critique stems from Freud's initial proposal of seduction theory, in 1896. Faced with many patients with symptoms of hysteria and obsessional neurosis, and some argue, responding to cues from Freud during analytic sessions (Masson, 1984), he theorized that these conditions were the result of repressed memories of early childhood abuse chiefly, it seems, by the father. The account that circulates is that faced with hostility from clinical colleagues and fearing opposition from society at large, along with a desire to protect the reputation of a generation of respectable Viennese men, Freud retreated from this provocative claim. Instead, he proposed that unconscious memories of abuse were likely to often be childhood fantasies on the grounds that the unconscious cannot differentiate between fact and fiction (Masson, 1984). The specific negative result of this claim is said to be the skepticism that victims of childhood abuse face when reporting such events to therapists (Rush, 1980). More generally, what might be described as Freud's privileging of psychic events over actual “observable” events as potential causes for psychopathology later in life has been challenged for example by Bowlby (1988) in his work on attachment and sharply differentiates Freud's work from a British empiricist tradition based on “observation” (Stänicke et al., 2020). Stänicke and colleagues argue that Freud's view was that neither psychic nor external reality could be known completely. The same “event” may have very different meanings for and effects on different individuals. Not only that but an individual may only at a later date come to attribute a traumatic or other meaning to an earlier event, a belated understanding, or *Nachträglichkeit* (Laplanche, 1985).

### 4.4 Freud, empathy, and the impossibility of loving your neighbor

One starting point for speculation on what Freud might say about empathy, caring, and compassion is to consider Isabel Menzies‐Lyth's classic study on social defenses against anxiety among nurses, published in 1959 and 1960 (Menzies, 1960). As a psychoanalyst working at London's Tavistock Institute of Human Relations, a center that specialized in studies of organizations, she was asked for help with a hospital's problem. “The senior staff” we are told, “felt that there was a danger of a complete breakdown in the system of allocating student nurses to front line work with patients, while also trying to train them effectively” (Lawlor, 2016). Menzies‐Lyth, following Freud's hermeneutic approach, as a mistress of suspicion, took this as a “presenting problem” masking a deeper psychic cause and set about to look for its origins. Her method, also like Freud, involved extensive talking with people, although she also carried out some under‐described observational work.

One of the ways that the organization of work in the study hospital was meant to reduce anxiety, claims Menzies‐Lyth but appeared to fail, was through various working arrangements for distancing nurses from patients by fragmenting the patient into a series of task‐based encounters. In the process, this attempt to evade anxiety thwarted any opportunity to gain satisfaction from exercising caring and compassion which nurses involved in the study said that they wished to do. Menzies‐Lyth believed that the same process that depersonalized the patient also depersonalized the nurse. The students, like the patients, were treated as “categories” such as “a second‐year,” and these categories associated them with an agglomeration of expected skills. She believed that the expression of any individuality in the work was discouraged, likewise the development of close relationships with patients. While this study was done not out of interest for the welfare of individual nurses but to avoid the spread of distress among the students and so stave off workforce instability, Menzies‐Lyth describes the deep ambivalence that caring evoked. Using Freud's description of infantile psychic life further developed by Klein (1975), as her conceptual framework, she concluded that the distress that the nurses experienced was connected to infantile anxieties aroused in the nurse by contact with seriously ill patients. As a Kleinian she saw these anxieties linked to the strong emotions of love, hate, and aggression and suggested that the nurse projects her own infantile fantasy into the workplace, experiencing the work as a deeply painful mixture of objective reality and fantasy. “The core of the anxiety [for nurses] lies in patient care and in the relationship with the patient” (Menzies‐Lyth, 1960 p. 100). Because student nurses had expected the hospital to be an environment in which they could find their own dependence supported she believed that they found the lack of personal care that they encountered particularly painful. Their witness to and involvement in caregiving to patients gave rise to a degree of jealousy and other strongly ambivalent feelings toward the patient. In other words, Menzies‐Lyth claimed that the students identified with the patients because they craved and perhaps expected to be recipients of care because, as neophytes, they shared vulnerability with the patients. The organization's defense against the resulting anxiety was essentially one of evasion and this unsuccessful attempt blocked the possibility for individual nurses to be helped to confront such anxiety and learn to deal with it more effectively. Without this, the empathy and caring that are idealized within the profession remain deeply ambivalent at a personal level.

It has been claimed (Tutton & Langstaff, 2015) that the publication of Menzies‐Lyth's work led eventually, after much resistance, to reforms to the organization of nursing work such as the rise of the concept of patient‐centered care and of Nursing Development Units (NDUs) in the UK with their particular organization of work. Some have described the brief period of the NDUs (the late 1980s and early 1990s) as a return to the profession's “humanistic base” in an increasingly mechanistic and science‐dominated health service (Maben et al., 2009). It is possible to ask, from a standpoint of suspicion, whether this more recent professional emphasis on empathy and caring is itself an unconscious way of searching for a way out of the nurse's experience of depersonalization while maintaining some sense of distance and control over work with patients. But could it be that assumptions about empathy, its naturalness, value, and even its possibility have been seriously under‐examined by the profession? A brief look at Freud's writing on “the neighbour” can allow us to consider this further.

In one of his later books, *Civilization, and its Discontents*, published in 1929 (Freud, 1963), Freud writes about the familiar biblical injunction to “love your neighbor as yourself.” This injunction first appears in the Old Testament book of Leviticus: “You shall not take vengeance or bear a grudge against the sons of your own people, but you shall love your neighbor as yourself: I am the Lord.” (Leviticus 19:18) and is later taken up by Jesus when asked by lawyers to identify the greatest of the Old Testament commandments (Matthew 22: 35‐40). Freud claims that it is impossible to obey—or at the very least the command is deeply problematic. For Freud repression turns everything on its head. What would be unacceptable to the conscious mind is actively excluded and becomes part of the always‐at‐work unconscious. Freud claims that aggressiveness is more primitive in terms of human development than love. We have to be socialized to love and be given these injunctions to “love our neighbor.” But my relationship with my neighbor, according to Freud, is more likely to be one of hatred and hostile rivalry. Moral behavior comes from culture and mutual hostility precedes this. It makes no sense to love the neighbor says Freud. And in any case, how could we love them in the same way that we love our kith and kin? Are we to understand the neighbor as an extension of ourselves, or the familial, someone *like* me? (Zizek et al., 2005 p. 6).

My relationship with my neighbor, or my patient, is enigmatic fundamentally because Freud and others argue, they are unknowable. For Emmanuel Levinas, the other person presents themself to us, when we encounter them, as “other,” rather than another version of ourselves that might be grasped and understood in its totality. We still have a responsibility toward them, to acknowledge them and their requirements, he argues, but their otherness, or alterity, remains and needs to be preserved (Levinas, 1969). From this perspective empathy, feeling with you, apparently intuitively, is problematic, for at least three reasons. First, there is something, we could say, imperialistic about my understanding of you. I can only know you from my own position and bring you into my system of understanding, or my profession's system whether that be a rational categorization or a belief in the possibility and value of empathy.^1^ But at best, I have a fantasy of who you are and what you are like. The danger of too strong a belief in this approach to empathy is the assumption that my experiences and emotional behaviors are the same as yours, that “*I know how you feel*.” For example, your eyes fill with tears when I ask about your mother because you never met her while my eyes fill with tears because I murdered mine and am still angry with her. Second, I do not really know how, why, or what often, I feel, at least if we take seriously the picture of the self divided into conscious and unconscious—as Freud suggests, let alone what another might be feeling. Third, I may find the prospect of radical aloneness too much to bear and seek comfort in infantile fantasies of deep connection and oneness with the humans I come across, for example, the patients that I have to care for. Nursing's talk of empathy could be problematic if it results in the unwitting involvement of patients in meeting nurses' own unconscious emotional needs—a reversal of Menzies‐Lyth's case study where now nurses might make efforts to encourage patients to express their own fears and vulnerabilities to their named nurse (Binnie, 1987).

Neuman (2010) makes a similar point in an investigation of the concept of empathy as a claimed function within the potentially therapeutic relationship of psychoanalysis. He takes as a starting point classic definitions of empathy from Heinz Kohut who suggested that “The best definition of empathy … is the capacity to think and feel oneself into the inner life of another person (Kohut, 1984, p. 82) [and that] Empathy is a basic endowment of man” and an “introspection” into others (Kohut, 1959, p. 144), a kind of “mind‐reading.” Against this understanding of empathy which is prevalent within nursing, Neuman proposes that the utterances and behavior—a cry or a grin perhaps—of another should be approached as an unfamiliar text. The first step, he claims, should be to project oneself not into “another mind” but into another language, the language—the symbolic structure—that the other is immersed in and employs. The danger of not doing this is that:

…what I might read is not the distant text but my own image as reflected through the other. My own self translocated into the other. This phantasy is actually a form of projective identification where the particularity of the other and his uniqueness is substituted for a replica of one's self particles (Neuman, 2010, p. 239).

This is a danger, or rather an unexplored complexity, that threatens nursing and nurses' claims about the centrality of empathy in their work and identity.

So to sum up this part of my argument, nurses' and nursing's strong identification with empathy, caring, and compassion, alongside the injunction on nurses to care, are problematic. According to Freud, Klein, and others, there is a developmentally earlier drive to hostility and separateness toward the other which the call to care has to cover over, as Menzies‐Lyth suggested. The attempt to fragment the potential relationship between nurse and patient of the 1950s hospital setting and the organization of work in NDUs of the 1980s and 90s can both be seen as ways to deal with this contradiction, first by evading it and later by incorporating it into a professionalizing project. The call and the claim to be empathetic is an impossibility if we, as divided subjects, do not even know ourselves, let alone the enigmatic other. The question remains then: how might we, or rather, how might Freud and his followers in suspicion, account for the identification with caring that is repeated in the profession, despite its problematic character?

## 5 DISCUSSION: WHICH IS THE NURSE, WHICH IS THE PATIENT?

This paper is not the first to problematize nursing's attachment to empathy, caring, and compassion, nor the first to apply psychoanalytic theory to aspects of nursing work. However in the spirit of a suspicion that sees a “presenting problem” as covering over—yet pointing to—an unconscious cause I would like to argue that an analysis of nursing's attachment to these notions, empathy, caring and compassion, can tell us something about the forces at work in the profession's unconscious. It can help us to understand the repetition of strong statements, both from individual nurses and by the profession as a whole, about the overriding value of caring coupled as it often has been with the expression of moral distress at organizational obstructions to its exercise (Jameton, 1984). Returning to my original intention which is the uncovering of “unthought,” and “disavowed presuppositions and consequences” of nursing texts on empathy, caring, and compassion, I would like to bring together two interlinked topics that emerged from my reading: the first is the ambivalence that Menzies‐Lyth believed afflicted students who entered nursing regarding who would be the giver, and who the receiver of care and their subsequent shock at the lack of support they received in dealing with the delivery of patient care. The second concerns the repetition of apparently fruitless investigations into caring noted by Paley. I would like to make the argument that each depends upon the other and that they are symptoms of unconscious processes.

I would like to consider the first topic–ambivalence about who is the carer and who the cared‐for. In 2013, I attended the Royal College of Nursing's annual congress in Liverpool in the UK. The congress was held at a unique time when the profession was subject to particular media and government criticism in the wake of the care scandals referred to earlier. This had a palpable effect on the atmosphere of the meeting which was more emotionally intense than in other years that I had attended. Approximately 3500 delegates join this congress. Platform speakers repeatedly referred to the pain and sense of injustice of being in a caring role but not being cared for oneself by managers or by the government or the public at large, more than one using poetry to express this. One speaker, a senior nurse, told a story of being a patient himself after a serious road accident and made the same point with a highly emotive story. Such expressions received standing ovations (see https://www.youtube.com/watch?v=XOCda6OiYpg). A debate on the nurse's role in caring for obese patients included a number of speakers using the podium to confess their shame, often tearfully, at failing to lose weight themselves and some were physically comforted by members of the audience who came to the stage to support them. During another debate about the evidence for the value of nursing, one speaker told the audience about an occasion when he had fed a patient with advanced dementia. The patient's condition demanded extreme care and patience on the nurse's part to avoid the patient choking and, he said, the process had taken an hour. At the end of this, the nurse told us that he saw the flicker of a smile on the patient's face and that he, the nurse, had felt a huge amount of satisfaction. It was the satisfaction *that he had experienced*, he told the audience, that was proof of the value of nursing work. All of these examples show, I think, nurses' identification with their patients, or identification as patients, their yearning to be cared for, and their distress when they feel that they are not. Neuman, above, describes one version, or misunderstanding, of the nature and challenge of empathy as the projection of one's own emotion and thoughts into the other. The nurse's strong identification as a carer can be understood as a fantasy of being the one who is cared for. So why might this expression be so often coupled with statements about moral distress and the impossibility of caring? As Menzies‐Lyth points out nurses face “heavy demands for pity, compassion and sympathy” (Menzies‐Lyth, 1960, p. 101) yet may find the tasks of nursing disturbing and particular patients difficult. This arouses, she goes on to suggest, guilt and anxiety because such feelings–disgust, impatience, and others–are not believed to be worthy of the profession. There is also something idealized about the character of the empathy, caring, and compassion that nurses identify with such that they can never match up to the ideal. As a result of the guilt and shame that this causes, nurses project this failure onto the outside in the form of the organization that they work within preventing their expression. Not only does the organization and its management constrain the expression of these qualities and ideals while simultaneously demanding them, but it also fails to care for nurses themselves in the midst of their own stressful and highly demanding work. Such complaints have been voiced over many decades by nurses (Traynor & Evans, 2014).

The second point that came to light concerns the repeated nature of research by nurses into caring and to this I would like to add the repetitive character of nurses' complaints mentioned above. From a Freudian point of view, repetition can be seen as a sign of the death drive in operation. Freud's theory of the death drive, as summarized earlier, is both biological and cosmic in character and he understood the compulsion to repeat as designed to achieve certain ends in these realms, that is, an attempt to experience some control over a traumatic stimulus retrospectively and to return to an inorganic state where nothing new occurs. Looking at the former type of explanation and with the *fort‐da* game of the toddler in mind, it has been argued that the repetition of disappearance followed by reappearance turns, for the child, a traumatic (unnameable and unmanageable) experience into an experience of loss which is something able to be symbolized and hence no longer traumatic (Lear, 2000). We could argue that nurses make a similar attempt to name the trauma inherent in nursing work, that is, terrible experiences without being supported, by repeating complaints about managers, or other workers, in other words, assertions of powerlessness. Whether this attempt (to symbolize the traumatic) is “successful” seems uncertain. Developments of this concept by Lacan and more recently Žižek can be applied in a more open‐ended way to elements of the human predicament including the repetition of nursing investigations into caring, and complaint, in a way that radically reframes the question about success or otherwise of these attempts. Lacan likens the death drive to automatism because it refers to a human tendency to repeatedly derail its own intentions, to be caught in a loop of repetition that brings a kind of satisfaction but not the satisfaction of achieving ends or achieving the aimed pleasure, but of the repetition of the failure to do so. This satisfaction is the “beyond” of Freud's pleasure principle, a potentially masochistic satisfaction that is at once the “cause” and the “result” of the death drive. Žižek writes of the death drive as leading not to annihilation as its name might suggest but to the realm of the “undead” an apparently Zombie‐like existence characterized by guilt and pain (Žižek, 2006), where actions are repeated long after they have any life or meaning.

To address a possible objection, there are other explanations for the proliferation of caring research that do not involve Zombies: organizational and other pressure to publish among them, and nursing research on caring is not the only area of research characterized by a lack of apparent progress. However, these things have other effects. The research as well as other statements about the centrality of caring to nursing form part of the backdrop to nursing work that most nurses would have an awareness of. Caring is being kept alive, in a Zombie‐like way, from a number of directions.

## 6 SO WHAT FOR THE PROFESSION? CAN THERE BE A TALKING CURE FOR NURSING?

Psychoanalytic insights suggest that caring roles can raise profoundly ambivalent issues for those who care. Freud's theories have been used to investigate nursing problems and the part played by unconscious forces in organizations. More fundamentally they can be used, as is the aim of this paper, to propose insights into puzzling or apparently contradictory observations about nursing such as the repetition of complaints about organizational constraints on care or of research into the character of caring. So, are there any implications for nursing and nurses of this critique? In some ways, the more radical the critique, the more radical the cure required. Marx, Freud, and Nietzsche did not envisage or provide an implementation program for their ideas save a total and violent transformation, of global society and its economic structures or systems of morality or of the psyche. And these levels are interconnected. The existence of the exploitation and commodification of emotion in nursing and emotional displays in the service of efficiency and capital show this clearly (Smith, 1992). Freud claimed to have founded a “talking cure” but there are many barriers to psychoanalytic work: an individual has got to seek out this talking cure by, at some level, acknowledging their responsibility for their suffering. An organization would have to do the same, as would a profession and both have competing interests that stand in the way of radical conversations. Menzies‐Lyth claims that her organizational involvement brought benefits for the organization of nursing work—without such a belief the whole enterprise of the Tavistock Institute would have no basis—but she is clear about the origin of the difficulties organizations and individuals face in understanding the source of their problems:

I think what may be happening is something like this. There is within the job situation a focus of deep anxiety and distress. Associated with this there is despair about being able to improve matters. The defensive system collusively set up against these feelings consists; first, in fragmentation of the core problem so that it no longer exists in an integrated and recognizable form consciously and openly among those concerned. Secondly, the fragments are projected onto bits of the ambience of the job situation, which are then consciously and honestly, but mistakenly, experienced as the problem about which something needs to be done, usually by someone else. Responsibility has also been fragmented and projected often into unknown others, “Them,” the authorities. One meets this same process frequently in psychoanalysis when a patient feels himself to be up against an intractable problem and believes he cannot manage the feelings associated with it. Such defensive reactions to institutional problems often mean the institution cannot really learn. The solutions tried before had failed, but they will work this time–as though there is a kind of magic about them. Effective resolution can only come when the institution, with or without the help of a consultant, can address itself to the heart of the matter and not only to its ambience, and introduce relevant changes there (Menzies‐Lyth, 1989, p. 30).

In other words, the “symptoms” that Freud and later Menzies‐Lyth detect always serve a purpose by protecting the individual or the organization from becoming aware of a profoundly disturbing problem. Nevertheless, psychoanalytic work is ultimately aimed at pointing the way to freedom from what can be painful and self‐destructive symptoms. Arriving at that point can, and probably inevitably does involve a disquieting journey where comforting certainties about ourselves and the value and meaning of what we do are profoundly questioned. A reviewer asked, “what might a Freudian “cure“ look like for nursing?” Does that question and my entering into it somehow conceal a desire that everything stays the same but just “magically” better? A thoroughly optimistic answer to this question for me raises an unrealistic scenario of large numbers of individuals and organizations engaged in psychodynamic work. However, carrying on with this thought experiment, one helpful effect might be that if empathy, caring and compassion can be taken out of the endless loop of transcendent possibility and crushing disappointment, nurses might have more time to understand their work as skills‐based (Nelson & Gordon, 2006) and for carefully listening to patients, though this would not deal with the anxiety that the work causes. But first, the rewards of the endless loop would need to be recognized.

## CONFLICT OF INTEREST

The author declares no conflict of interest.

## Data Availability

Data sharing is not applicable to this article as no datasets were generated or analyzed during the current study

## ENDNOTE

1 Not everyone would agree with this assessment of absolute otherness. For a summary of the difference between the ideas of Levinas and Gabriel Marcel, the latter of whom believed that “love” could enable a knowing of the other, see Treanor (2006, ch. 1).
